# Supplementary material for: Prolactin-mediates a lactation-induced suppression of arcuate kisspeptin neuronal activity necessary for lactational infertility in mice
Source: eLife. 2025 Jan 17;13:RP94570. doi: 10.7554/eLife.94570 (PMC11741520; doi:10.7554/eLife.94570)
Supplement: Supplementary file 1. [file elife-94570-supp1.docx]

**Supplementary Table 1.** Statistics table. Abbreviations for tables below: DF = degrees of freedom; mc = multiple comparison; CI = 95% confidence interval; MW U = Mann Whitney U; MEA = Mixed effect analysis (fixed effects (type III)), RM = repeated measures. Supp = supplementary data figure.

| **Fig.** | **Description** | **Statistical analysis** | | | | |
| --- | --- | --- | --- | --- | --- | --- |
| 1C | RP3V Kisspeptin immunoreactivity in *Prlr*^lox/lox^/*Camk2a*^Cre^ mice |  | **p** | **DF** | **CI** | **R^2^** |
|  | Interaction | Two-way ANOVA | 0.0034 | 1 |  |  |
|  | Genotype | Two-way ANOVA | 0.0251 | 1 |  |  |
|  | State | Two-way ANOVA | 0.1380 | 1 |  |  |
|  | *Prlr*^lox/lox^:NL vs. *Prlr*^lox/lox^:Lactating | Tukey’s mc | 0.0100 | 23 | 5.175 to 44.95 |  |
|  | *Prlr*^lox/lox^:NL vs. *Prlr*^lox/lox^/*Camk2a*^Cre^:NL | Tukey’s mc | 0.9429 | 23 | -17.79 to 26.81 |  |
|  | *Prlr*^lox/lox^:NL vs. *Prlr*^lox/lox^/*Camk2a*^Cre^:Lactating | Tukey’s mc | 0.9228 | 23 | -24.38 to 15.39 |  |
|  | *Prlr*^lox/lox^:Lactating vs. *Prlr*^lox/lox^/*Camk2a*^Cre^:NL | Tukey’s mc | 0.0565 | 23 | -41.55 to 0.4412 |  |
|  | *Prlr*^lox/lox^:Lactating vs. *Prlr*^lox/lox^/*Camk2a*^Cre^:Lactating | Tukey’s mc | 0.0010 | 23 | -47.97 to -11.15 |  |
|  | *Prlr*^lox/lox^/*Camk2a*^Cre^:NL vs. *Prlr*^lox/lox^/*Camk2a*^Cre^:Lactating | Tukey’s mc | 0.6409 | 23 | -30.00 to 11.99 |  |
| 1D | Arcuate Kisspeptin immunoreactivity in *Prlr*^lox/lox^/*Camk2a*^Cre^ mice | Unpaired two-tailed t test | 0.0020 | 14 | 0.6692 to 2.424 | 0.5052 |
| 1E | *Prlr*^lox/lox^/*Camk2a*^Cre^ do not show lactational diestrus |  | **p** | **X^2^** | **DF** |  |
|  | Percentage of mice showing estrus | Log rank (Mantel-Cox) test | <0.0001 | 42.37 | 1 |  |
| 1 Supp1 | Proportion of kisspeptin neurons showing *Prlr* deletion using RNAscope in *Prlr*^lox/lox^/*Camk2a*^Cre^ mice |  | **p** | **R^2^** | **CI** |  |
|  | B: RP3V | Unpaired two-tailed t test | <0.0001 | 0.8740 | -55.02 to 28.69 |  |
|  | D: ARC | Unpaired two-tailed t test | 0.0009 | 0.8123 | -69.72 to 8.860 |  |
| 1 Supp2 | *Prlr*^lox/lox^/*Camk2a*^Cre^ maternal and gestational phenotyping |  | **p** | **R^2^** | **CI** | **MW U** |
|  | A: gestational weight gain | Unpaired two-tailed t test | 0.1291 | 0.1113 | -3.076 to 0.4219 |  |
|  | B: gestation length | Mann Whitney test | 0.2682 |  |  | 45.50 |
|  | C: number of live pups | Unpaired two-tailed t test | 0.7479 | 0.005282 | -4.037 to 2.946 |  |
|  | D: litter weight gain |  |  |  |  |  |
|  | Time | MEA | <0.0001 |  |  |  |
|  | Genotype | MEA | 0.3833 |  |  |  |
|  | Time x genotype | MEA | 0.3282 |  |  |  |
|  | Day 3 lactation | Šídák's mc | >0.9999 |  | -3.740 to 4.115 |  |
|  | Day 4 lactation | Šídák's mc | 0.9993 |  | -3.365 to 4.490 |  |
|  | Day 5 lactation | Šídák's mc | 0.9863 |  | -2.965 to 4.890 |  |
|  | Day 6 lactation | Šídák's mc | 0.9525 |  | -2.690 to 5.165 |  |
|  | Day 7 lactation | Šídák's mc | 0.6938 |  | -1.953 to 5.903 |  |
|  | Day 8 lactation | Šídák's mc | 0.619 |  | -1.821 to 6.112 |  |
| 2B | Effect of mifepristone treatment on LH pulse frequency in *Prlr*^lox/lox^/*Camk2a*^Cre^ mice |  | **p** | **DF** | **CI** |  |
|  | Interaction | Two-way ANOVA | 0.2807 | 1 |  |  |
|  | Genotype | Two-way ANOVA | 0.0024 | 1 |  |  |
|  | Treatment | Two-way ANOVA | 0.8558 | 1 |  |  |
|  | *Prlr*^lox/lox^:Veh vs. *Prlr*^lox/lox^:Mif | Tukey’s mc | 0.7922 | 27 | -0.9804 to 1.980 |  |
|  | *Prlr*^lox/lox^:Veh vs. *Prlr*^lox/lox^/*Camk2a*^Cre^:Veh | Tukey’s mc | 0.3861 | 27 | -2.355 to 0.6054 |  |
|  | *Prlr*^lox/lox^:Veh vs. *Prlr*^lox/lox^/*Camk2a*^Cre^:Mif | Tukey’s mc | 0.1487 | 27 | -2.765 to 0.3002 |  |
|  | *Prlr*^lox/lox^:Mif vs. *Prlr*^lox/lox^/*Camk2a*^Cre^:Veh | Tukey’s mc | 0.0758 | 27 | -2.765 to 0.3002 |  |
|  | *Prlr*^lox/lox^:Mif vs. *Prlr*^lox/lox^/*Camk2a*^Cre^:Mif | Tukey’s mc | 0.0223 | 27 | -2.765 to 0.3002 |  |
|  | *Prlr*^lox/lox^/*Camk2a*^Cre^:Veh vs. *Prlr*^lox/lox^/*Camk2a*^Cre^:Mif | Tukey’s mc | 0.9188 | 27 | -1.890 to 1.175 |  |
| 2C | Effect of mifepristone treatment on mean LH in *Prlr*^lox/lox^/*Camk2a*^Cre^ mice |  | **p** | **DF** | **CI** |  |
|  | Interaction | Two-way ANOVA | 0.8572 | 1 |  |  |
|  | Genotype | Two-way ANOVA | 0.0023 | 1 |  |  |
|  | Treatment | Two-way ANOVA | 0.8457 | 1 |  |  |
|  | *Prlr*^lox/lox^:Veh vs. *Prlr*^lox/lox^:Mif | Tukey’s mc | 0.9928 | 27 | -0.09533 to 0.1164 |  |
|  | *Prlr*^lox/lox^:Veh vs. *Prlr*^lox/lox^/*Camk2a*^Cre^:Veh | Tukey’s mc | 0.1237 | 27 | -0.1947 to 0.01699 |  |
|  | *Prlr*^lox/lox^:Veh vs. *Prlr*^lox/lox^/*Camk2a*^Cre^:Mif | Tukey’s mc | 0.1463 | 27 | -0.1980 to 0.02112 |  |
|  | *Prlr*^lox/lox^:Mif vs. *Prlr*^lox/lox^/*Camk2a*^Cre^:Veh | Tukey’s mc | 0.0715 | 27 | -0.2053 to 0.006462 |  |
|  | *Prlr*^lox/lox^:Mif vs. *Prlr*^lox/lox^/*Camk2a*^Cre^:Mif | Tukey’s mc | 0.0875 | 27 | -0.2086 to 0.01059 |  |
|  | *Prlr*^lox/lox^/*Camk2a*^Cre^:Veh vs. *Prlr*^lox/lox^/*Camk2a*^Cre^:Mif | Tukey’s mc | >0.9999 | 27 | -0.1092 to 0.1100 |  |
| 2  Supp 2A | Mifepristone functional dose response trial |  | **p** | **X^2^** | **Z** |  |
|  |  | Chi-squared test | 0.0072 | 6 | 2.449 |  |
| 2  Supp  2B | Mifepristone has no effect on litter weight gain |  | **p** | **DF** |  |  |
|  | Time x Genotype and treatment | Two-way RM ANOVA | 0.5322 | 6 |  |  |
|  | Time | Two-way RM ANOVA | <0.0001 | 2 |  |  |
|  | Genotype and treatment | Two-way RM ANOVA | 0.8811 | 3 |  |  |
|  | Subject | Two-way RM ANOVA | <0.0001 | 22 |  |  |
| 4B | Kisspeptin activity across different reproductive states |  | **p** | **DF** | **CI** |  |
|  | SE frequency across states | MEA | 0.0040 |  |  |  |
|  | NLvs. Day 4 pregnancy | Tukey’s mc | 0.0828 | 5 | -0.1356 to 2.052 |  |
|  | NL vs. Day 14 pregßnancy | Tukey’s mc | 0.0586 | 5 | -0.05175 to 2.552 |  |
|  | NL vs. Day 18/19 pregnancy | Tukey’s mc | 0.9828 | 3 | -7.819 to 5.962 |  |
|  | NL vs. Day 7 lactation | Tukey’s mc | 0.0912 | 4 | -0.2517 to 2.752 |  |
|  | NL vs. Day 14 lactation | Tukey’s mc | 0.6584 | 3 | -1.598 to 2.931 |  |
|  | NL vs. Day 18 lactation | Tukey’s mc | 0.9638 | 4 | -1.661 to 2.461 |  |
|  | NL vs. 24h after weaning | Tukey’s mc | >0.9999 | 3 | -3.097 to 2.847 |  |
|  | NL vs. Return to estrous cycles | Tukey’s mc | 0.9941 | 3 | -4.126 to 3.292 |  |
|  | NL vs. OVX | Tukey’s mc | 0.0009 | 5 | -4.955 to -2.100 |  |
|  | Day 4 pregnancy vs. Day 14 pregnancy | Tukey’s mc | 0.5635 | 5 | -0.3793 to 0.9626 |  |
|  | Day 4 pregnancy vs. Day 18/19 pregnancy | Tukey’s mc | 0.7685 | 3 | -9.378 to 5.605 |  |
|  | Day 4 pregnancy vs. Day 7 lactation | Tukey’s mc | 0.6396 | 4 | -0.5180 to 1.101 |  |
|  | Day 4 pregnancy vs. Day 14 lactation | Tukey’s mc | 0.4286 | 3 | -1.026 to 0.4430 |  |
|  | Day 4 pregnancy vs. Day 18 lactation | Tukey’s mc | 0.2948 | 4 | -1.579 to 0.4622 |  |
|  | Day 4 pregnancy vs. 24h after weaning | Tukey’s mc | 0.1309 | 3 | -2.649 to 0.4820 |  |
|  | Day 4 vs. Return to estrous cycles | Tukey’s mc | 0.1718 | 3 | -3.598 to 0.8484 |  |
|  | Day 4 pregnancy vs. OVX | Tukey’s mc | <0.0001 | 5 | -5.683 to -3.290 |  |
|  | Day 14 pregnancy vs. Day 18/19 pregnancy | Tukey’s mc | 0.6513 | 3 | -9.510 to 5.153 |  |
|  | Day 14 pregnancy vs. Day 7 lactation | Tukey’s mc |  | | | |
|  | Day 14 pregnancy vs. Day 14 lactation | Tukey’s mc | 0.0343 | 3 | -1.092 to -0.07436 |  |
|  | Day 14 pregnancy vs. Day 18 lactation | Tukey’s mc | 0.0092 | 4 | -1.380 to -0.3202 |  |
|  | Day 14 pregnancy vs. 24h after weaning | Tukey’s mc | 0.0072 | 3 | -2.072 to -0.6781 |  |
|  | Day 14 pregnancy vs. Return to estrous cycles | Tukey’s mc | 0.0333 | 3 | -3.106 to -0.2271 |  |
|  | Day 14 pregnancy vs. OVX | Tukey’s mc | <0.0001 | 5 | -5.877 to -3.679 |  |
|  | Day 18/19 pregnancy vs. Day 7 lactation | Tukey’s mc | 0.7328 | 2 | -9.856 to 14.21 |  |
|  | Day 18/19 pregnancy vs. Day 14 lactation | Tukey’s mc | 0.9290 | 2 | -12.04 to 15.23 |  |
|  | Day 18/19 pregnancy vs. Day 18 lactation | Tukey’s mc | 0.9317 | 3 | -6.119 to 8.776 |  |
|  | Day 18/19 pregnancy vs. 24h after weaning | Tukey’s mc | 0.9749 | 2 | -7.900 to 9.507 |  |
|  | Day 18/19 pregnancy vs. Return to estrous cycles | Tukey’s mc | 0.9990 | 2 | -8.964 to 9.988 |  |
|  | Day 18/19 pregnancy vs. OVX | Tukey’s mc | 0.4480 | 3 | -9.320 to 4.121 |  |
|  | Day 7 lactation vs. Day 14 lactation | Tukey’s mc | 0.0382 | 3 | -1.112 to -0.05439 |  |
|  | Day 7 lactation vs. Day 18 lactation | Tukey’s mc | 0.0325 | 3 | -1.578 to -0.1222 |  |
|  | Day 7 lactation vs. 24h after weaning | Tukey’s mc | 0.0412 | 2 | -2.620 to -0.1304 |  |
|  | Day 7 lactation vs. Return to estrous cycles | Tukey’s mc | 0.1060 | 2 | -4.140 to 0.8071 |  |
|  | Day 7 lactation vs. OVX | Tukey’s mc | 0.0002 | 4 | -5.887 to -3.669 |  |
|  | Day 14 lactation vs. Day 18 lactation | Tukey’s mc | 0.8130 | 3 | -1.405 to 0.8715 |  |
|  | Day 14 lactation vs. 24h after weaning | Tukey’s mc | 0.1642 | 2 | -2.285 to 0.7020 |  |
|  | Day 14 lactation vs. Return to estrous cycles | Tukey’s mc | 0.2779 | 2 | -3.864 to 1.698 |  |
|  | Day 14 lactation vs. OVX | Tukey’s mc | 0.0026 | 3 | -5.734 to -2.655 |  |
|  | Day 18 lactation vs. 24h after weaning | Tukey’s mc | 0.1662 | 3 | -1.362 to 0.3122 |  |
|  | Day 18 lactation vs. Return to estrous cycles | Tukey’s mc | 0.1035 | 3 | -1.892 to 0.2583 |  |
|  | Day 18 lactation vs. OVX | Tukey’s mc | 0.0016 | 4 | -5.475 to -2.380 |  |
|  | 24h after weaning vs. Return to estrous cycles | Tukey’s mc | 0.6226 | 3 | -1.237 to 0.6537 |  |
|  | 24h after weaning vs. OVX | Tukey’s mc | 0.0080 | 3 | -5.192 to -1.614 |  |
|  | Return to estrous cycles vs OVX | Tukey’s mc | 0.0231 | 3 | -5.470 to -0.7521 |  |
| 4C | Kisspeptin activity across different reproductive states |  | **p** | **DF** | **CI** |  |
|  | SE amplitude across states | MEA | NA |  |  |  |
|  | NL vs. Day 4 | Tukey’s mc | 0.9571 | 2 | -1.626 to 1.981 |  |
|  | NL vs. Day 18/19 | Tukey’s mc | 0.2608 | 3 | -0.6762 to 2.086 |  |
|  | NL vs. Day 14 | Tukey’s mc | 0.0851 | 3 | -0.1362 to 1.365 |  |
|  | NL vs. Day 18 | Tukey’s mc | 0.1766 | 4 | -0.2966 to 1.467 |  |
|  | NL vs. 24h after weaning | Tukey’s mc | 0.4700 | 3 | -0.9997 to 2.178 |  |
|  | NL vs. Return to estrous cycles | Tukey’s mc | 0.4475 | 3 | -0.8540 to 1.911 |  |
|  | NL vs. OVX | Tukey’s mc | 0.9938 | 5 | -0.9886 to 0.7359 |  |
|  | Day 4 vs. Day 18/19 | Tukey’s mc |  |  |  |  |
|  | Day 4 vs. Day 14 | Tukey’s mc | 0.3617 | 1 | -2.590 to 3.464 |  |
|  | Day 4 vs. Day 18 | Tukey’s mc | 0.3714 | 1 | -2.498 to 3.313 |  |
|  | Day 4 vs. 24h after weaning | Tukey’s mc | 0.4348 | 1 | -3.091 to 3.915 |  |
|  | Day 4 vs. Return to estrous cycles | Tukey’s mc | 0.2644 | 1 | -1.387 to 2.089 |  |
|  | Day 4 vs. OVX | Tukey’s mc | 0.9098 | 2 | -2.837 to 2.229 |  |
|  | Day 18/19 vs. Day 14 | Tukey’s mc | 0.7069 | 2 | -0.5763 to 0.3960 |  |
|  | Day 18/19 vs. Day 18 | Tukey’s mc | 0.1985 | 3 | -0.3267 to 0.08725 |  |
|  | Day 18/19 vs. 24h after weaning | Tukey’s mc | 0.6222 | 2 | -0.6603 to 0.4286 |  |
|  | Day 18/19 vs. Return to estrous cycles | Tukey’s mc | 0.3505 | 2 | -0.7040 to 0.3514 |  |
|  | Day 18/19 vs. OVX | Tukey’s mc | 0.1494 | 3 | -2.103 to 0.4405 |  |
|  | Day 14 vs. Day 18 | Tukey’s mc | 0.9929 | 3 | -0.3118 to 0.2526 |  |
|  | Day 14 vs. 24h after weaning | Tukey’s mc | 0.5747 | 2 | -0.1375 to 0.08624 |  |
|  | Day 14 vs. Return to estrous cycles | Tukey’s mc | 0.4387 | 2 | -0.3880 to 0.2157 |  |
|  | Day 14 vs. OVX | Tukey’s mc | 0.3306 | 3 | -2.375 to 0.8931 |  |
|  | Day 18 vs. 24h after weaning | Tukey’s mc | >0.9999 | 3 | -0.3977 to 0.4056 |  |
|  | Day 18 vs. Return to estrous cycles | Tukey’s mc | 0.9118 | 3 | -0.3693 to 0.2562 |  |
|  | Day 18 vs. OVX | Tukey’s mc | 0.1809 | 4 | -1.793 to 0.3702 |  |
|  | 24h after weaning vs. Return to estrous cycles |  | 0.8456 | 3 | -0.3448 to 0.2238 |  |
|  | 24h after weaning vs. OVX | Tukey’s mc | 0.2874 | 3 | -2.185 to 0.7540 |  |
|  | Return to estrous cycles vs. OVX | Tukey’s mc | 0.2975 | 3 | -2.023 to 0.7136 |  |
| 5C | NL vs day 18/19 pregnancy frequency of SE |  | **p** | **R^2^** | **CI** |  |
|  |  | Paired two-tailed t test | 0.4063 | 0.2364 | -2.646 to 4.945 |  |
| 5D | NL vs day 18/19 pregnancy relative SE amplitude |  | **p** | **R^2^** | **CI** |  |
|  |  | Paired two-tailed t test | 0.0141 | 0.8989 | -1.606 to -0.3816 |  |
| 6A | *Prlr*^lox/lox^/*Kiss1*^Cre^ do not show lactational diestrus |  | **p** | **X^2^** | **DF** |  |
|  | Percentage of mice showing estrus | Log rank (Mantel-Cox) test | <0.0001 | 38.02 | 1 |  |
| 6  Supp  1 | Proportion of kisspeptin neurons showing *Prlr* deletion using RNAscope in *Prlr*^lox/lox^/*Kiss1*^Cre^ |  | **p** | **R^2^** | **CI** |  |
|  | B: ARC | Unpaired two-tailed t test | <0.0001 | 0.9300 | -64.27 to -44.46 |  |
|  | C: correlation of day of estrus and average density of *Prlr* on *Kiss1*-expressing cells (per animal) | Simple linear regression | 0.3262 | 0.1951 |  |  |
| 6 Supp 2 | *Prlr*^lox/lox^/*Kiss1*^Cre^ maternal and gestational phenotyping |  | **p** | **R^2^** | **CI** | **MW U** |
|  | A: gestational weight gain | Unpaired two-tailed t test | 0.6361 | 0.003694 | -0.9985 to 1.622 |  |
|  | B: gestation length | Mann Whitney test | 0.7670 |  |  | 471.5 |
|  | C: number of live pups | Unpaired two-tailed t test | 0.4522 | 0.009296 | -0.5930 to 1.315 |  |
|  | D: litter weight gain | | | | | |
|  | Time x Genotype | Two-way RM ANOVA | 0.6404 |  |  |  |
|  | Time | Two-way RM ANOVA | <0.0001 |  |  |  |
|  | Genotype | Two-way RM ANOVA | 0.9014 |  |  |  |
|  | Subject | Two-way RM ANOVA | <0.0001 |  |  |  |
|  | Day 3 lactation | Šídák's mc | >0.9999 |  | -5.407 to 3.653 |  |
|  | Day 4 lactation | Šídák's mc | >0.9999 |  | -5.204 to 3.857 |  |
|  | Day 5 lactation | Šídák's mc | >0.9999 |  | -5.159 to 3.902 |  |
|  | Day 6 lactation | Šídák's mc | >0.9999 |  | -5.105 to 3.956 |  |
|  | Day 7 lactation | Šídák's mc | >0.9999 |  | -4.976 to 4.084 |  |
|  | Day 8 lactation | Šídák's mc | >0.9999 |  | -4.226 to 4.834 |  |
|  | Day 9 lactation | Šídák's mc | >0.9999 |  | -4.757 to 4.304 |  |
|  | Day 10 lactation | Šídák's mc | >0.9999 |  | -4.650 to 4.410 |  |
|  | Day 11 lactation | Šídák's mc | >0.9999 |  | -5.154 to 3.907 |  |
|  | Day 12 lactation | Šídák's mc | >0.9999 |  | -5.242 to 3.819 |  |
|  | Day 13 lactation | Šídák's mc | >0.9999 |  | -4.936 to 4.125 |  |
|  | Day 14 lactation | Šídák's mc | >0.9999 |  | -4.979 to 4.081 |  |
|  | Day 15 lactation | Šídák's mc | >0.9999 |  | -4.776 to 4.284 |  |
|  | Day 16 lactation | Šídák's mc | >0.9999 |  | -4.152 to 4.909 |  |
|  | Day 17 lactation | Šídák's mc | >0.9999 |  | -4.216 to 4.845 |  |
|  | Day 18 lactation | Šídák's mc | 0.9912 |  | -2.717 to 6.343 |  |
|  | Day 19 lactation | Šídák's mc | >0.9999 |  | -4.182 to 4.878 |  |
|  | Day 20 lactation | Šídák's mc | >0.9999 |  | -4.752 to 4.309 |  |
